# Impact of Vitamin D on Physical Efficiency and Exercise Performance—A Review

**DOI:** 10.3390/nu11112826

**Published:** 2019-11-19

**Authors:** Michał Wiciński, Dawid Adamkiewicz, Monika Adamkiewicz, Maciej Śniegocki, Marta Podhorecka, Paweł Szychta, Bartosz Malinowski

**Affiliations:** 1 Department of Pharmacology and Therapeutics, Faculty of Medicine, Collegium Medicum in Bydgoszcz, Nicolaus Copernicus University, M. Curie 9, 85-090 Bydgoszcz, Poland; 2 Department of Neurosurgery, Neurotraumatology and Paediatric Neurosurgery, Collegium Medicum in Bydgoszcz, Nicolaus Copernicus University, 85-094 Bydgoszcz, Poland; 3 Department of Geriatrics, Collegium Medicum in Bydgoszcz, Nicolaus Copernicus University, 85-094 Bydgoszcz, Poland; 4 Department of Plastic, Reconstructive and Aesthetic Surgery, Collegium Medicum in Bydgoszcz, Nicolaus Copernicus University, 85-094 Bydgoszcz, Poland

**Keywords:** 25(OH)D, VDR, VDBP, athlete, physical efficiency, exercise performance, vitamin D deficiency

## Abstract

Vitamin D deficiency amongst athletes and the general population seems to be a prominent problem. The most recognized role of vitamin D is its regulation of calcium homeostasis; there is a strong relationship between vitamin D and bone health. Moreover, its concentrations are associated with muscle function and immune response in both the general and athletic populations. Vitamin D level is strongly connected with the presence of VDRs (vitamin D receptors) in most human extraskeletal cells. Expression of multiple myogenic transcription factors enhancing muscle cell proliferation and differentiation is caused by an exposure of skeletal muscles to vitamin D. The aim of this review is to summarize current understanding of the significance of vitamin D on exercise performance and physical efficiency, as well to analyze the impact of vitamin D on multiple potential mechanisms. More high-quality research studies, considering free 25(OH)D as a better marker of vitamin D status, the baseline level of 25(OH)D and multiple pathways of vitamin D acting and usage in athletes are required.

## 1. Introduction

Vitamin D is a group of steroid compounds demonstrating the same biological activity, mainly associated with affecting calcium phosphate economy. It occurs in nature in the forms of vitamin D2 (ergocalciferol) and vitamin D3 (cholecalciferol). Vitamin D2 is produced by fungi and yeast by UVB-exposure of ergosterol (provitamin D2), therefore, ergocalciferol is found in sun-dried mushroom products and in plant-based products (as a result of contamination with fungi) [1,2]. Accordingly, vitamin D2 is generally known as a plant form of vitamin D, whereas vitamin D3 is produced by UVB-exposure of 7-dehydrocholesterol (provitamin D3) in the skin of many vertebrate animals, including humans. Cholecalciferol can be provided by consuming animal products like fatty fish (mackerel, salmon, sardines), fish oil, eggs and liver or, in smaller amounts, meat, dairy products, offal and poultry [3]. Nevertheless, over 90% of vitamin D in the human body is formed in the skin tissue influenced by sunlight. Provitamin D (7-dehydrocholesterol) is converted under ultraviolet B (UVB) rays to previtamin D3 and ultimately to vitamin D3 [4,5]. It is certain that 25(OH)D can be increased by vitamin D supplementation, proper nutrition and exposing uncovered skin to sun radiation (sun angle should be over 30°) [6]. There are many other factors that decrease the level of vitamin D, such as obesity (adiposity may decrease bioavailability of 25-hydroxyvitamin D) [7], liver failure, senectitude, darker skin tone or sunscreen use [8,9,10]. Smoking cigarettes also has a significant negative influence on the level of vitamin D [11]. Beyond modifiable factors there are dimensions such as genetic determinants, individual variations [12] or age that are not modifiable [13]. The relationship between gender and vitamin D deficiency or 25(OH)D level is inconsistent and elaborate in non-athletes. Verdoia et al. observed significantly lower 25(OH)D levels in females (female 14.5 ± 10.9 ng/mL vs. male 15.9 ± 9.5 ng/mL, *p* = 0.007), which might be strongly related to the older age of women participating in the study comparing to men (*p* < 0.001). Moreover, a higher rate of vitamin D deficiency occurred especially in post-menopausal women. [14]. On the contrary, the study of AlQuainz et al. identified a higher prevalence of vitamin D deficiency among males when compared to females (72.0% (*n* = 695) vs. 64.0% (*n* = 1191)) [15]. Likewise, the study of Johnson et al., conducted on morbidly obese patients, showed that male patients had significantly lower mean 25(OH)D concentrations than female patients (50.0 ± 22.0 nmol/L versus 53.6 ± 22.4 nmol/L (*p* = 0.001)) and a higher rate of vitamin D deficiency than women (56% vs. 47%; *p* < 0.001) [16]. Gender differences can be related with both known (for example, more men smoke cigarettes and are less likely than women to avoid high-fat foods, women wear hijabs covering the whole body) and unknown habits. The differences by sex may be due to variations in body fat percentage and body size [17]. The decrease in vitamin D bioavailability is thought to be due to sequestration of vitamin D in adipose tissue after cutaneous synthesis or dietary intake, although the exact mechanism is not yet known. However, the study of Halliday et al. showed no difference in vitamin D status depending on sex in athletes (*p* = 0.10). Any correlations between body mass, BMI or body fat percentage adjusted by sex were also not found in athletes. The reason for a lack of a significant correlation between vitamin D status and adiposity in this study is most likely explained by the reduced body fat range of the athlete population. Moreover, different sport disciplines were studied in relation to male and female athletes [18].

Vitamin D insufficiency seems to be a very prominent problem amongst athletes and the general population [19,20]. The evidence available suggests that there is a cause for concern regarding the vitamin D concentration of athletes depending on sun exposure. The results of a huge meta-analysis of 23 studies with 2313 athletes demonstrated that 56% of them had vitamin D inadequacy. Vitamin D insufficiency significantly varied by latitude, also, it was higher during winter and at the beginning of the spring season [21] and for those athletes doing indoor sport activities [22]. The difference between outdoor and indoor athletes is noticeable in the study of Aydin et al., which showed the prevalence of vitamin D deficiency in 59% of outdoor athletes and 64% of indoor athletes. The study was conducted during winter season [4]. Peeling et al. found significantly lower vitamin D status in indoor (90 ± 28 nmol/L) compared to outdoor (131 ± 35 nmol/L) athletes (*p* = 0.0001). Considering the predominant training environment during the summer season, 50% trained predominately indoors (31 gymnastics, 5 diving) and 30.6% trained predominately outdoors (10 sailing, 7 field hockey, 3 athletics, 2 rowing) [23]. Halliday et al. documented that 25(OH)D concentrations changed across time (*p* = 0.001) and averaged 49.0 ± 16.6, 30.5 ± 9.4, and 41.9 ± 14.6 ng/mL in the fall, winter, and spring, respectively, in a group of 41 collegiate athletes (12 indoor (wrestling, swimming, basketball) and 29 outdoor athletes (football, soccer, cross-country or track and field, and cheerleading/dance)). Vitamin D levels were significantly higher in outdoor compared with indoor athletes in the fall (53.1 ± 17.4 vs. 39.3 ± 8.9 ng/mL, *p* = 0.013); in the winter or spring, vitamin D concentrations were higher in outdoor athletes, but these differences were not statistically significant. Of the athletes studied, 75.6% in the fall, 15.2% in the winter, and 36.0% in the spring had optimal 25(OH)D status (40 ng/mL was the cut-off for optimal status). Neither total vitamin D intake nor intake from food alone differed across time or with sex or training location (*p* < 0.05) [18].

## 2. Metabolism of Vitamin D and Mechanisms of Vitamin D Receptor

The activity of vitamin D is highly connected with cytochrome P450 enzymes which are responsible for both activation (25-hydroxylase, 1α-hydroxylase) and deactivation (24-hydroxylase) of vitamin D. Firstly, 25(OH)D is produced in the liver, from D2 or D3 with the contribution of CYP2R1 (25-hydroxylase), and then in the kidneys, the biologically active form of vitamin D— 1,25(OH)2D—is synthesized by CYP27B1 (1α-hydroxylase) from 25(OH)D. Inactivation of vitamin D goes gradually by 24-hydroxylase that forms inactive metabolites 24,25-dihydroxyvitamin D and 1,24,25-trihydroxyvitamin D from 25(OH)D and 1,25(OH)2D, respectively. In the end, CYP3A4 degrades them [24]. The enzymes mentioned above are magnesium-dependent; optimal magnesium concentration, depending on baseline level of 25(OH)D, can be helpful to reaching proper 25(OH)D target concentration [25]. 

The biological function of vitamin D, in principle 1,25-dihydroxyvitamin D—the active metabolite—is unspecifically mediated by binding to a single vitamin D receptor (VDR) that is expressed in several tissues [26,27]. Moreover, direct influence of 1,25(OH)2D is also possible through specific plasma membrane receptors [28].

A VDR consists of three main domains: the C-domain that binds DNA, the E-domain that connects with a ligand and the activating F-domain. The vitamin D receptor is closely connected to RXR (retinoic acid receptor); the 5^′^ arm of the nucleotide sequence of VDRE acts with RXR, and for VDR, it is the 3^′^ arm. As it is a ligand-dependent transcription factor, it binds VDR and RXR along with some other proteins and activators (SARC1-3 and DRIP205), creating a transcription complex at the VDREs so the phosphorylation is initiated [29].

It seems to be significant to mention the vitamin D-binding protein (VDBP) which is produced by hepatic parenchymal cells’ glycoprotein; its main function is linking almost 90% of 25(OH)D and 1,25-dihydroxyvitamin D3 circulating in serum. Out of the total 25(OH)D, albumin binds approximately 10% and less than 1% remains free (albumin-bound and “free” are the only bioavailable forms). Black men have lower levels of VDBP than white men, having lower mean concentration of total serum 25(OH)D at the same time (Black 25.0 ± 14.7 vs. White 37.4 ± 14.0; *p* < 0.001) [30,31].

## 3. Vitamin D in Sport Performance

Due to vitamin D insufficiency in the population, many studies undertake the topic of correlation between vitamin D insufficiency and impaired sport performance, defined as skeletal muscle function. Vitamin D is one of the most regularly used dietary supplements amongst athletes worldwide and is the number one supplement taken by athletes with a physical impairment [32,33]. Close et al. found a significantly positive correlation between vitamin D3 supplementation (5000 IU per day for 8-weeks) and the improvement of musculoskeletal performance, especially in vertical jump height and 10-m sprint times. The study was a placebo-controlled trial conducted on 61 male athletes coming from different sports (soccer, rugby, flat and hunt jockeys) and 30 controls who were apparently healthy non-athletic individuals. It was found that 62% of the athletes had a baseline level of total serum 25(OH)D below 50 nmol/L and also that 73% of the controls were 25(OH)D-deficient or had inadequate 25(OH)D level. Total serum 25(OH)D significantly increased from baseline in the vitamin D treated group of athletes [34]. 

Moreover, Książek et al. showed that there is a positive correlation between vitamin D levels and sport performance. They enrolled 25 representatives of the Polish National judo team who were in the general preparation period and were performing almost the same exercises. It was found that 80% of the athletes were vitamin D deficient, with less than 30 ng/mL 25(OH)D in the baseline. The results of the study presented a statistically positive correlation between 25(OH)D concentration and the power of vertical jump. There was also significant correlation with left hand grip strength and total work for both the right and left lower extremities during extension [35].

Rowing is a discipline where most of the energy is supplied via aerobic metabolism similar to other endurance types of sports. There is a correlation between aerobic power and high maximum oxygen consumption (VO_2_max) where the transport of oxygen plays a key role. It is coherent that improving hematological levels is a way to enhance rowers’ athletic performance. Thirty-six elite male rowers were randomly separated into a control group (CG, *n* = 18, height 181.05 ± 3.39 cm, body mass 77.02 ± 7.55 kg) and a group supplemented with 3000 IU of vitamin D/day for eight weeks (VD3G, *n* = 18, height: 179.70 ± 9.07, body mass: 76.19 ± 10.07 kg). Mielgo-Ayuso et al. intended to check if vitamin D supplementation was a suitable factor appealing to hematological and iron metabolism profile. All of the athletes had baseline suboptimal 25(OH)D concentrations (26.24 ± 8.18 ng/mL) and the tests were carried out in the spring months (April–June) in Spain. Statistically, a significant increase in the serum concentration of 25(OH)D in the VD3G after 8 weeks (T2) was observed (T1 26.24 ± 8.18 ng/mL vs. T2 48.12 ± 10.88 ng/mL; *p* < 0.001). In the CG (control group) no statistically significant changes were noticed (T1 30.76 ± 6.95 ng/mL vs. T2 35.14 ± 7.96 ng/mL; *p* = 0.056). Significant differences in the group-by-time interaction of hemoglobin (*p* = 0.009), hematocrit (*p* = 0.019) and transferrin (*p* = 0.007) between the two groups through the study were observed. Moreover, in the CG, a significant decrease (*p* < 0.05) in hemoglobin during the study (T1 15.54 ± 0.88 ng/mL vs. T2 15.09 ± 0.82 ng/mL), and a significant increase (*p* < 0.05) in VD3G in transferrin levels over 8 weeks (T1: 254.22 ± 20.69 vs. 270.44 ± 20.08 mg/dL) were observed. Although, in both groups, all body composition measures (body mass, BMI, sum 4 skinfolds, fat mass and free fat mass) presented significant differences during the study (*p* < 0.05), the group-by-time interaction of them between groups did not show any significant difference (*p* > 0.05). All rowers followed the same training program (the average weekly hours of training were 15 during the study). These results show that vitamin D supplementation affects hematological parameters and can enhance aerobic power by increasing VO_2max_. However, authors did not give any information as to if the rowers were at the beginning or in the middle of their training season, which would have a relevant impact on the interpretation of results. Moreover, the study group of 36 rowers divided into two groups seems to be too small and limiting [36]

Analysis of the results of Koundourakis et al.’s research on 67 Caucasian male professional soccer players of three Greek football teams showed some interesting findings that not only exposure to UVB but also reduction of training stress may have a good influence on serum vitamin D levels. Vitamin D concentration was found to be increased significantly following the six-week off-season period (47.24 ± 13.50 ng/mL) compared to baseline (34.41 ± 7.08 ng/mL) while at the same time, all measured performance parameters decreased. Moreover, the study demonstrated a significant positive linear association between 25(OH)D level and muscle strength as evaluated by squat and countermovement jumps, sprinting ability (10- and 20-m) and aerobic capacity—VO_2max_—in professional soccer players. According to this study, vitamin D levels probably play a significant supportive role in sport performance [37].

The study of Jastrzębska et al. was conducted on 36 soccer players (age: 17.5 ± 0.6 years, body mass 71.3 ± 6.9 kg, BMI 22.2 ± 1.8 kg/m^2^) who were members of a sports school (Poland) that educated the highly talented youth. Players were divided into two groups: the placebo one, *n* = 16 and the experimental one, *n* = 20 (which was vitamin D3 supplemented). The selection was done deliberately into homogenous groups and even the number of players of the same field position was equal. Initial 25(OH)D levels did not differ between supplemented and placebo groups (48.5 ± 8.6 vs. 47.5 ± 16.2 mmol/L, *p* = 0.817). In the supplemented group, the plasma 25(OH)D level increased significantly after the intervention (initial 48.5 ± 8.6 mmol/L, after intervention 106.3 ± 26.6 mmol/L, *p* < 0.0001), while in the placebo group, only non-significant changes were observed (initial 47.5 ± 16.2 mmol/L, after intervention 43.5 ± 16.7 mmol/L, *p* = 0.228). The following variables improved significantly compared to the placebo group: maximal running velocity (V_max_, *p* = 0.017), running velocity at lactate threshold (V_LT_, *p* < 0.0001), V_LT_/V_max_ (*p* = 0.000006), maximal heart rate (*p* = 0.0001), physical work capacity (*p* < 0.0001) and maximal oxygen uptake (*p* < 0.0001) [38].

In the above studies, the subjects were supplemented with daily doses of vitamin D. Wyon et al. separated 22 judo athletes into two groups and supplemented one with a one-time dose of 150,000 IU. Nevertheless, the treatment group demonstrated a significant increase in muscle strength between days 1 and 8 (*p* = 0.01) [39].

On the other hand, a number of published reports fail to document any statistically significant impact of increasing vitamin D level on exercise performance [30]. A recent placebo-controlled double-blind study conducted on 36 top Polish young junior soccer players, aged 17.5 ± 0.6 years, did not demonstrate statistically significant difference between the placebo and the supplemented with vitamin D groups. After eight weeks of the same high-intensity soccer training activity, it was found that there was just a little positive difference in physical activity indicators (time–motion parameters and heart rate) in comparison to placebo group. Baseline level of 25-hydroxyvitamin D did not differ significantly between supplemented (48.5 ± 8.6 nmol/L) and non-supplemented groups (47.5 ± 16.2 nmol/L) [40]. 

Furthermore, Orysiak et al. conducted a cross-sectional study on a group of 50 adolescent male ice hockey players. They found that vitamin D insufficiency is highly prevalent in this group, nevertheless, there was no positive correlation between serum 25(OH)D concentration and isometric muscle strength, vertical jump performance, or repeated sprint ability (RSA) after adjusting for age, training experience, fat mass, fat free mass and height. Exercise performance was evaluated using isometric strength measures of upper and lower extremities, vertical jump performance and RSA [41].

A study on 22 rugby players supplemented with 50 000 IU of cholecalciferol (equivalent to 3570 IU/day) or placebo once every two weeks over 11–12 weeks demonstrated significant increase of serum 25(OH)D concentrations in the treatment group compared to placebo (*p* < 0.001). Performance in five of the six tests, including the primary outcome variable of 30-m sprint time, did not differ between the vitamin D supplemented and placebo groups (*p* > 0.05). Only performance on the weighted reverse-grip chin up was significantly higher in players receiving vitamin D compared with placebo (*p* = 0.002) [42].

Todd et al. observed similar findings in a research on 43 Gaelic football players, who received twelve-week daily supplementation with 3000 IU of vitamin D during wintertime. The vitamin D supplementation had no significant effect on VO_2_ max (*p* = 0.375), skeletal muscle and lung function, or vertical jump height when compared to the placebo group (*p* = 0.797). It also had no significant effect on left or right handgrip strength (*p* = 0.146 and *p* = 0.266, respectively). Although the concentration of 25(OH)D increased (*p* = 0.006), there was no correlation between total 25(OH)D and measures of physical performance (*p* > 0.05) [43]. The characteristics of the included studies are shown in Table 1.

Whilst many researchers succeeded at showing that supplementation of vitamin D in athletes improved markers of muscle function and physical performance, equally many studies failed to show a significant correlation. The baseline of vitamin D concentration may underline these discrepancies [44]. A number of reports conclude the best enhancement after vitamin D treatment occurs to athletes with critically low baseline status of 25(OH)D (under 30 ng/mL). Improvements in athletic performance when the baseline is over 30 ng/mL are less expressed or even unnoticeable [45]. 

Hollis et al. conducted a study on two separate populations: the first, 93 surfers and skateboarders from Hawaii who received significant sun exposure (3 or more hours per day on 5 or more days per week for at least the preceding 3 months); the second, subjects from a lactation study who were separated into two vitamin D supplementation groups, group 1 (placebo) with 400 IU vitamin D_3_/day or group 2 (high-dose supplemented) which received 6400 IU vitamin D_3_/day for 6 months. Both populations achieved similar concentrations of circulating 25(OH)D levels: surfers and skateboarders (11–71 ng/mL) and high-dose supplemented group of women (12–77 ng/mL). This suggests either/or product–substrate inhibition of 25-hydroxylase. Optimal nutritional vitamin D status may occur when approaching equimolar concentrations of circulating vitamin D3 and 25(OH)D. At this point, the V_max_ of the enzyme appears to be achieved. It is important to note that as humans live today, the 25-hydroxylase operates well below its V_max_ because of chronic substrate (vitamin D) deficiency. This study suggests that a minimum circulating level of 25(OH)D should be >80 nmol (32 ng/mL). It seems to be a significant concentration for both non-athletes and athletes [46]. Cholecalciferol does not begin to be routinely stored in fat and muscle tissue for future use until 25(OH)D levels reach 40–50 ng/mL. At lower levels, the initial 25-hydroxylation in the liver usually follows first-order mass action kinetics, and the reaction is not saturable. That is, at levels below 40–50 ng/mL, the body diverts most or all of the ingested or sun-derived vitamin D to immediate metabolic needs, signifying chronic substrate starvation. According to research, a recommended level of 25(OH)D for athletes seems to be not less than 40 ng/mL (100 nmol/L) because at this concentration the muscle and fat starts to store vitamin D for future use [47].

## 4. Potential Mechanisms of Vitamin D Impact on Exercise Performance

Analyzing many research papers, it appears that the consideration of the impact of vitamin D on athletic performance as a single-track mechanism is incomplete. Since the 1970s, several reports have described reversible morphological changes in skeletal muscles of severe vitamin D-deficient patients expressing type II muscle fiber atrophy. The changes yielded with vitamin D supplementation. Vitamin D induces myogenesis and muscle protein synthesis causing an increase of percentage of fast twitch muscle cell (type II fibers). This type of fiber is responsible for high power output, fast muscle contraction and muscle development [48]. 

Vitamin D receptors (VDR) seem to play a significant role in muscle regeneration after injury when the expression of VDR increases. A local presence and activation of 1α-hydroxylase in injured muscle fibers lets the muscle synthesize an active form—1,25(OH)2D [49]. The signaling axes of VDR mediation are not well defined. However, smad3 (mothers against decapentaplegic homolog 3) that implicates the bone morphogenetic protein (BMP) and transforming growth factor (TGF-β1), Src (proto-oncogene tyrosine-protein kinase Src), phosphoinositide 3 kinase (PI3K), and cAMP responsible element-binding protein (CREB) cascades seem to have significant predictive meaning in muscle progenitor differentiation and regeneration of muscle following damage [30]. The role of vitamin D in intracellular pathways of muscle in, e.g., inducing mitochondrial function, was documented. Mitochondrial oxidative phosphorylation is the main resource of ATP for skeletal muscle contraction in severe vitamin D-deficient subjects. Sinha et al. proved that cholecalciferol therapy raised the mean serum status of 25(OH)D and simultaneously caused an improvement in maximal oxidative phosphorylation and a reduced recovery half-time of phosphocreatine [50]. 

Antinozzi et al. evaluated the in vitro effect of elocalcitol, a non-hypercalcemic VDR agonist, on the biomolecular metabolic machinery of human fetal skeletal muscle cells (Hfsmc) vs. insulin. The in vivo vitamin D status and VDR muscular expression were investigated in inflammatory myopathic subjects (31 Caucasian patients with dermatomyositis, polymyositis or polymyositis associated with other connective tissue diseases). Hfsmc, an in vitro cell system previously validated, was treated with elocalcitol or insulin for comparison and analyzed for gene expression of glucose transporter (Glut, Glut3 and Glut1), translocation of Glut4 and localization of Flotillin-1, Caveolin-1 and Caveolin-3. All proteins associated with cell membrane reorganization/vesicle formation during exocytosis/cell trafficking; activation of mammalian target of rapamycin (mTOR), protein kinase B (PKB/AKT), extracellular-signal-regulated kinases (ERK1/2) and eukaryotic translation initiation factor 4E-binding protein 1 (4EBP1), all insulin-responsive paths were involved in glucose metabolism. VDR mRNA and protein expression have been also investigated in Hfsmc. Elocalcitol exerted an insulin-like effect, promoting GLUT4 re-localization in Flotillin-1, Caveolin-3 and Caveolin-1 positive sites and mTOR, AKT, ERK, 4E-BP1 activation; it enhanced IL-6 myokine release. Elevated concentrations of IL-6 during muscle contraction send a signal to the liver that glycogen levels are critically low. This activity helps to control metabolic homeostasis. VDR agonists, such as elocalcitol, may be a therapeutic tool for skeletal muscle integrity/function maintenance. Presented investigation shows that the improvement of vitamin D status—i.e., by supplementation with less- or non-hypercalcemic VDR agonist like elocalcitol—might be a helpful tool to counteract detrimental processes in skeletal muscle, not only in myopathy or diabetes patients, but also in athletes. More studies are required to affirm if this kind of treatment may be useful for physically active people to improve physical abilities, endurance and diabetic control [51].

## 5. Summary

According to the review, it is undoubtedly important to conduct another high-quality research study. One of the main aspects that should be taken into account is important evidence suggestive that free (bioavailable) 25(OH)D can turn out to be a better marker of vitamin D status. Many researchers do not take under consideration that athletes may require an increased supply of vitamin D to fulfill muscle metabolism requirements because of potential pathways of vitamin D usage. Significant debate seems to be needed to determine and standardize a classification of vitamin D deficiency.

Summarizing the results of this review, there are many incompatible reports about correlation between vitamin D supplementation and exercise performance for athletes. It is still uncertain if vitamin D supplementation shall be definitely recommended, however it seems to give beneficial effects while incorporating the baseline level of vitamin D status in planning studies and analyzing the results. 

## Figures and Tables

**Table 1:** Vitamin D and sport performance.

Author	Study Group	Vitamin D Doses	Baseline 25(OH)D	Endpoint 25(OH)D	Results
Close et al., 2013 [34]	*n* = 61 UK soccer players, rugby players, flat and hunt jockeys	5000 IU/day for 8 weeks vs. placebo	D3T: 29 ± 25 nmol/L CG: 53 ± 29 nmol/L	D3T: 103 ± 25 nmol/L CG: 74 ± 24 nmol/L	Increase in 10-m sprint times (*p* = 0.008) and vertical jump (*p* = 0.008) in the vitamin D group vs. placebo
Książek et al., 2018 [35]	*n* = 25 Polish judoists	no supplementation	17.4 ± 5.2 ng/mL; 80% of subjects were <30 ng/mL	-	Positive correlation between 25(OH)D and left hand grip strength (*p* < 0.05), level and power of vertical jump (*p* < 0.05) and total work in left and right lower extremity during extension.
Koundourakis et al., 2014 [37]	*n* = 67 Greek soccer players	No supplementation, 6-week off-season	34.41 ± 7.08 ng/mL	47.24 ± 13.50 ng/mL after 6 weeks	Positive correlation between 25(OH)D level and squat jump (*p* < 0.001), countermovement jump (*p* < 0.001), VO_2max_ (*p* < 0.001). Negative correlation between 25(OH)D level and 10-m sprint time (*p* < 0.001), 20-m sprint time; (*p* < 0.001).
Jastrzębska et al., 2016 [38]	*n* = 36 Polish soccer players	5000 IU/day for 8 weeks vs. placebo	D3T: 48.5 ± 8.6 mmol/L CG: 47.5 ± 16.2 mmol/L	D3T: 106.3 ± 26.62 mmol/L CG: 43.5 ± 16.7 mmol/L	Significant improvement of maximal running capacity, running velocity at lactate threshold, maximal heart rate, physical work capacity and maximal oxygen uptake vs. placebo.
Wyon et al., 2015 [39]	*n* = 22 UK adult judoka athletes	150,000 IU once for 8 days vs. placebo	D3T: 13.16 ± 3.75 ng/mL CG: 16.33 ± 2.73 ng/mL	D3T: 16.76 ± 3.21 ng/mL CG: 16.33 ± 2.56 ng/mL	Significant increase in muscle strength between days 1 and 8 vs. placebo group (*p* = 0.01).
Skalska et al., 2019 [40]	*n* = 36 Polish young soccer players	5000 IU/day for 8 weeks vs. placebo	D3T: 48.5 ± 8.6 nmol/L CG: 47.5 ± 16.2 nmol/L	D3T: 106.3 ± 26.6 nmol/L CG: 43.5 ± 16.9 nmol/L	Comparing the supplemented and un-supplemented groups, no significant differences were found in any of the analyzed indicators.
Orysiak et al., 2018 [41]	*n* = 50 Polish ice hockey players	No supplementation	30.3 ± 14.9 ng/mL; 62% of subjects were <30 ng/mL	-	No correlation between 25(OH)D concentration and isometric muscle-strength, vertical jump performance and repeated sprint ability (*p* > 0.05)
Fairbairn et al., 2017 [42]	*n* = 57 New Zealand professional rugby players	50,000 IU once every 2 weeks for 11–12 weeks	D3T: 94 ± 18 nmol/L CG: 95 ± 17 nmol/L	D3T: 114 ± 19 nmol/L CG: 80 ± 21 nmol/L	No significant difference between supplemented and placebo groups (*p* > 0.05)
Todd et al., 2016 [43]	*n* = 43 Gaelic football players	3000 IU/ day for 12 weeks vs. placebo	D3T: 47.37 ± 13.3 nmol/L CG: 43.1 ± 22.0 nmol/L	D3T: 83.68 ± 32.98 nmol/L CG: 49.22 ± 25.40 nmol/L	Supplementation of vitamin D had no significant effect on VO_2max_, skeletal muscle function and lung function.

D3T—group supplemented with vitamin D; CG—control group; *n*—number of subjects.
